# Enhancing Wireless Network Efficiency with the Techniques of Dynamic Distributed Load Balancing: A Distance-Based Approach

**DOI:** 10.3390/s24165406

**Published:** 2024-08-21

**Authors:** Mustafa Mohammed Hasan Alkalsh, Adrian Kliks

**Affiliations:** 1 Department of Mobile Networks, Nokia Solutions and Networks, 54-130 Wroclaw, Poland; 2 Institute of Radiocommunications, Poznan University of Technology, 60-965 Poznan, Poland; adrian.kliks@put.poznan.pl; 3 Department of Computer Science, Electrical and Space Engineering, Luleå University of Technology, 971 87 Lulea, Sweden

**Keywords:** congested cells, ultra-dense networks, mobility management, handover, B5G networks

## Abstract

The unique combination of the high data rates, ultra-low latency, and massive machine communication capability of 5G networks has facilitated the development of a diverse range of applications distinguished by varying connectivity needs. This has led to a surge in data traffic, driven by the ever-increasing number of connected devices, which poses challenges to the load distribution among the network cells and minimizes the wireless network performance. In this context, maintaining network balance during congestion periods necessitates effective interaction between various network components. This study emphasizes the crucial role that mobility management plays in mitigating the uneven load distribution across cells. This distribution is a significant factor impacting network performance, and effectively managing it is essential for ensuring optimal network performance in 5G and future networks. The study investigated the complexities associated with congested cells in wireless networks to address this challenge. It proposes a Dynamic Distance-based Load-Balancing (DDLB) algorithm designed to facilitate efficient traffic distribution among contiguous cells and utilize available resources more efficiently. The algorithm reacts with congested cells and redistributes traffic to its neighboring cells based on specific network conditions. As a result, it alleviates congestion and enhances overall network performance. The results demonstrate that the DDLB algorithm significantly improves key metrics, including load distribution and rates of handover and radio link failure, handover ping-pong, and failed attached requests.

## 1. Introduction

The massive growth in the number of connected smart devices and applications that require variant quality of service (QoS) and quality of experience (QoE) requirements has resulted in a huge demand for mobile broadband services with high data rates. To cope with this demand, mobile network capacity and performance must be increased. The 5G new radio (NR) network standard offers the utilization of high-band spectrum such as millimeter wave bands, which can increase the network capacity and provide high coverage to users [1]. The 5G NR technology operates in a wide range of frequencies, balancing between coverage and data rates. Sub-6 GHz bands (4.5 GHz to 6 GHz) provide good coverage and better signal propagation, making them suitable for wider areas. However, data rates are moderate in these bands. In contrast, mmWave bands (24.25 GHz to 100 GHz) offer significantly higher data rates due to their large bandwidth [2,3]. The use of mmWave is one of the most promising and effective solutions for 5G and beyond (B5G) mobile networks. By using mmWave, wireless networks can achieve high data rates, as mmWave signals can carry a much larger amount of data, even with lower-order modulation schemes for reliable transmission, due to the enormous bandwidths that are available in these bands [4]. Looking ahead, 6G research is actively exploring even higher frequencies and novel technologies to further enhance data rates and network capacity. Potential areas of focus include terahertz (THz) bands exceeding 100 GHz and beyond, as well as advancements in areas like intelligent resource management and network slicing for optimized performance [5]. Nevertheless, mmWave presents certain difficulties for mobile communication as well because of its high sensitivity to abrupt channel changes and problems with obstructions from different structures, including trees and buildings. This may result in severe channel interruptions due to the signal distortion, as well as a substantial path loss when the signal intensity dramatically drops with distance [6].

Therefore, heterogeneous networks, or HetNets, which combine multiple radio access technologies, hold the key to the future of the network beyond 5G. In addition to macro cells, HetNets make use of small cells, which are the most widely used mmWave operating solution due to their low cost, low power consumption, and short-range radio-access nodes. Small cells are low-power base stations (such as picocells, femtocells, microcells, and relays) that offer cellular coverage in areas with high user traffic, whereas macro-cells are high-power base stations that cover broader areas. Despite this, and due to the power disparity between cell types, users tend to initiate connections to high-power cells that have the strongest signal than other cells. Using HetNets is one of the most significant ways to meet the demands on mobile networks to increase their capacity and performance. Users can switch between different types of cells based on their needs, which results in improved data rates and coverage [7].

Small cells can be deployed within the coverage areas of macro cells to enhance network performance and capacity in areas with high or imbalanced user demand. However, implementing HetNets presents significant difficulties with interference management, load balancing (LB), and mobility management (MM). One of the main challenges is the handover (HO) process, which is the technique that enables the mobile user equipment (UE) to switch its connection from one cell to another [8]. Several parameters that can control the HO process such as time-to-trigger (TTT), HO margin (HOM), cell frequency offset, and cell individual offset are called HO control parameters (HCPs), which affect both the QoS and the HO performance of the network [9]. As a result, it is critical to research how HCPs affect HO performance and determine the ideal parameters under various circumstances.

Another major challenge that affects network performance while maintaining connection stability is handling overload (OL) scenarios when they arise, particularly when some network cells are overloaded and have all of their resources reserved while other cells are not being used to their full potential. Neighboring cells can share the load from a loaded cell using their available resources when the cell becomes overloaded. The cell of HetNets with OL state results in an unequal data rate distribution, which degrades user experience and imposes inconsistency in the QoS and QoE. Therefore, mechanisms to offload the congested cells and balance the load dynamically are needed to ensure the efficient use of system resources. These mechanisms should be designed to distribute the traffic among contiguous cells proportionally based on their load conditions while providing satisfactory QoS for all users, especially those at the cell edge.

This study introduces a mobility controller model that periodically interacts with any cell within the network that reaches a relatively higher load than the average load. This average load is determined based on the load conditions among the surrounding cells to ensure maximum cell resource utilization for small-cell network deployments. The model utilizes an effective algorithm that guarantees the best offset values, which assures a successful HO process in several scenarios and applies them instantly. Based on the load distribution in the loaded cell, the load conditions in the adjacent cells, and the channel conditions determined by the position of the UE, the model dynamically modifies the HCP configuration that is configured by the operator. A simulation, compliant with 3GPP standards, was utilized to evaluate the model. Its results were compared with existing methods, focusing on maintaining a low handover ping-pong (HOPP) rate and decreasing both handover failure (HOF) and radio link failure (RLF) rates, thereby improving network performance. Moreover, it lowers the number of OL-related failed connection attempts for UEs inside the closest cell’s coverage, where the UEs are connected to the next best cell, which offers less favorable coverage circumstances than the cell that is fully loaded. Lastly, a review of the challenges and possible directions for this study are presented. Let us note that this paper addresses a real-life problem where mobile network operators have to manage extremely increased traffic over specific periods with the infrastructure originally designed for another scale.

The rest of the paper is organized as follows. Section 2 provides background on MM scenarios and related works, whereas Section 3 addresses the system model and the proposed DDLB algorithm for handling congested scenarios. Before Section 5 concludes this study, Section 4 presents the simulation scenario and its related performance evaluation by configuring the simulation scenario and results discussion.

## 2. Background

The functionality of MM is considered the backbone of seamless connectivity in wireless networks wherever users move within the network. It ensures that the connection of users is maintained and that their services are in excellent shape. The importance of MM could reach dropping calls and cutting off the data transfers, which would cause the user experience, overall, to suffer significantly. This becomes more critical in 6G networks, where applications like autonomous vehicles demand ultra-reliable and low-latency connections. Without efficient and smart MM mechanisms, such applications could face critical delays or even safety risks [10,11]. Furthermore, MM plays a substantial role in managing the ever-increasing number of connected devices in future wireless networks, ensuring efficient resource allocation and optimal network performance for all users.

The first subsection of this section provides a brief background on MM in 5G networks and its evolution in 6G, highlighting the impact of congested scenarios on network performance, while the second subsection provides the related works that focus on the cell load and its challenges in MM in 5G HetNets.

### 2.1. Mobility Management in Future Wireless Networks

**Figure 1:**
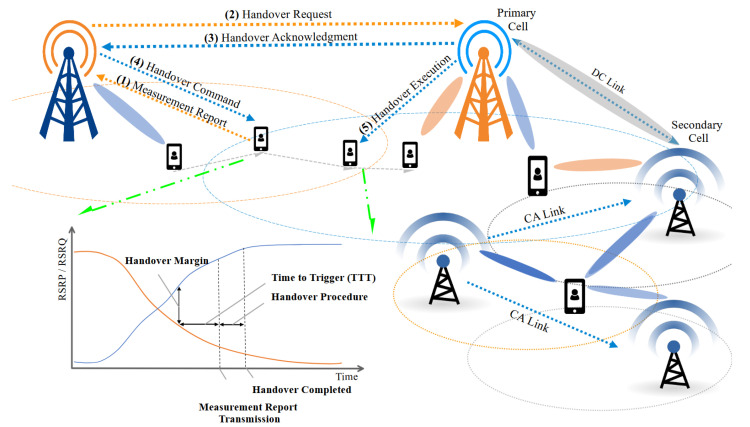
Some critical functionalities of Mobility Management in wireless networks.

Important improvements are introduced by 5G networks in the wireless network world such as increasing network capacity, enhancing user experience, and supporting wider user applications than previous generations. These applications are grouped under variant categories such as Enhanced Mobile Broadband (eMBB), Ultra-Reliable Low-Latency Communication (URLLC), and Massive Machine-Type Communication (mMTC). Thus, MM is one of the vital aspects of the 5G improvements, which ensures seamless connectivity during users’ mobility while crossing different cells or base stations (BSs) [12]. Thus, MM plays a crucial role in coordinating several key functionalities of wireless networks. These functionalities include the following, as illustrated in Figure 1:

- Attach and Detach Management: To establish a call or activate a data session, the UE initiates the attach procedure toward the network. This process involves registering the UE with the network, obtaining an identity, and negotiating service parameters. While ending a call or deactivating a data session, the UE initiates the detach process to notify the network and release resources. These procedures ensure efficient resource management and enable the network to track the active UEs and maintain service continuity [13].
- Location Management: The UEs location is continuously monitored by MM by the tracking cell associations and periodic signaling. This provides the network with a real-time understanding of the UEs location and activity, which aids efficient resource allocation and service delivery [14].
- Handover Management: When a UE with a Radio Resource Control (RRC) connected state moves from one cell to another, MM seamlessly transfers the ongoing data session (e.g., call, data, etc.) to the new cell. This process ensures a smooth transition and minimizes latency, preventing noticeable disruptions for the user [8].
- Context-Aware Management: As networks develop towards greater intelligence, MM incorporates context-aware management. By using specific information about network conditions, user behavior, and application requirements, MM can prioritize traffic and optimize resource allocation for specific use cases to enhance network performance and user experience [15].
- Network Slicing Management: 5G and future networks introduce network slicing, which virtually divides the physical network into customizable slices that serve specific service requirements. MM facilitates seamless handover between slices, allowing users to switch based on their needs (e.g., high bandwidth for video streaming or low latency for autonomous vehicles) while maintaining connectivity [16].
- Dual Connectivity (DC) Management: DC is an innovative technology that allows UEs to connect to two adjacent cells simultaneously [17]. This can be beneficial in areas with limited coverage or for high data rate requirements. MM plays a crucial role in managing DC cases by efficiently distributing traffic between the two connections, ensuring optimal utilization of available resources, and maximizing network performance. Furthermore, ensuring seamless transitions when UE moves between areas with and without DC support maintain active connections and minimize service disruptions.
- Carrier Aggregation (CA) Management: CA allows for UEs to combine multiple frequency bands simultaneously. This effectively increases the overall bandwidth available to the UE, enhancing the data rate [17]. MM also plays a critical role in managing CA where it intelligently selects and combines suitable frequency bands based on network conditions and user requirements, maximizing the benefits of CA. Furthermore, it ensures the aggregated channels are seamlessly transferred to the new cell during the HO process when UE moves between cells, maintaining data rates and minimizing service disruptions.

However, 6G networks target a lower latency and higher reliability than 5G to allow for applications like real-time control of robots and haptic feedback in virtual reality [18]. Furthermore, 6G is expected to support higher speeds than 5G, catering to applications like hyper-fast downloads and next-generation transportation systems [19]. It is also expected to leverage Ultra-Dense HetNets to increase network capacity and improve coverage, especially in urban areas. The high bandwidth and low latency of 6G are expected to enable the widespread adoption of drones for various applications such as delivery services, infrastructure inspection, and public safety. Also, they could be used as movable cells to support unexpected gathering scenarios that put the network in an overloaded state or provide coverage to zones in which temporary coverage is needed [20]. Thus, MM requires further development to cope with the demands for more efficient handover techniques and optimized resource allocation. This will give the ability to manage the applications in real time and handle various cell types within Ultra-Dense HetNets, high mobility speed users, and unique mobility patterns of drones.

While MM struggles with seamless connectivity, congestion cases in some or all cells of the network can turn the cells into an overloaded state. This significantly disturbs the network effectiveness, therefore increasing HO delays and RL failure when a UE moves from one cell to another. During an overloaded state, congested cells (close to the UE) may not be able to accommodate new connections efficiently. The UE might need to wait longer for the overloaded cell to accept the handover request, leading to disruptions in service. Moreover, if the overloaded cell consistently rejects handover requests due to insufficient resources, it can result in a higher RL failure rate, indicating an unsuccessful handover. This can force the UE to connect to a farther, less congested cell, impacting performance [21]. On the other hand, when a UE cannot connect to the closest cell because of overload, it might connect to the second closest cell if it is available and not overloaded. However, this “second closest cell” might also be overloaded due to increased traffic received from the UEs rejected by the first overloaded cell. This creates a cascading effect, where an overloaded state in one cell can cause an overload in neighboring cells. This also contributes to the “closest loaded cell” problem. UEs might prioritize connecting to the closest cell, even if it is overloaded, due to a shorter signal path and potentially lower power consumption. This further exacerbates congestion in the overloaded cell and increases the likelihood of RL failure rate. The overloaded cells struggle to handle the high volume of data traffic from numerous connected UEs. This can increase the packet loss rate due to insufficient resources in the overloaded cell, resulting in missing information and disruptions in services like video streaming or video calls [22].

Understanding the negative impacts of congested cell scenarios boosts the necessity of developing MM strategies to mitigate that impact and ensure efficient handovers, resource allocation, and overall network performance. Sharing the load partially with the neighboring cells that have the lowest load among the surrounding cells could reduce the negative impact on network performance. Therefore, this article proposes a DDLB algorithm that reacts periodically on the cell that is relatively loaded compared with its neighboring cells, taking into consideration the importance of achieving the seamless connectivity and optimal user experience promised by future wireless networks. The algorithm shows valuable enhancements in the network performance when compared with a standard network performance. It is achieved by reducing the number of failed attach attempts of UEs while establishing a connection with the network with the best available cell, minimizing the number of HOF rates, maintaining the lowest PP effect rates, and ensuring lower periods of OL states.

### 2.2. Related Works

LB is significantly important in 5G and 6G networks due to the robustness and reliability requirements of various applications such as streaming video, cloud computing, and virtual reality. It can help to optimize network performance and ensure that resources are used more efficiently. However, researchers often overlook the potential benefits of improving MM strategies to mitigate the impact of OL scenarios immediately during their occurrence. The understanding of how MM techniques can be adapted or improved to reduce the impact of congested cells on the performance of the network is a challenging gap. Ensuring a seamless handover, efficient load balancing, and the continuity of service delivery are the critical factors that need to be taken into consideration. Addressing this gap is essential for guaranteeing user mobility and maintaining service quality for applications that rely heavily on real-time connectivity in future networks. To balance the loads between the cells more efficiently, several studies have been carried out in the literature.

Simulations by [23] attempted to demonstrate the effectiveness of basic Load Balancing (LB) algorithms. LB mechanisms automatically tune configuration parameters, which increases cell edge throughput and reduces call blocking. The implementation of this technique in mobile networks optimizes a utility function that dynamically splits data across multiple access networks, regardless of application or QoS requirements.

A study by [24] proposed a traceable analytical network model that reduces Inter-Cell Interference (ICI) by using a combination of a reverse frequency assignment scheme and user correlation based on cell expansion. The researchers analyzed how this combination, with or without selective small cell base stations (sBSs), impacts coverage probability and user data rates in 5G networks. Their results showed that using reverse frequency allocation (RFA) improves coverage and user rates by mitigating ICI for unloaded macro-cell base stations (mBSs). They also found that RFA can improve performance by enhancing Channel Reuse Efficiency (CRE). Moreover, deploying selective sBSs can significantly improve coverage and user rates without requiring extra resources. However, the authors in [25] proposed a cell selection algorithm for load balancing in HetNets. This method uses two SINR thresholds to ensure user throughput. The algorithm creates two candidate lists for femtocells if the received SINR value is below the first threshold. The first is based on measured Received Signal Strength (RSS) values, while the second uses predicted RSS values. The final target femtocell is chosen based on the number of available physical resource blocks (PRBs) and pilot signal strength. If no suitable femtocell is available, users are forced to connect to the macro cell. Simulations showed that this approach improves user throughput compared to the traditional Max-SINR method but may not perform well for users with high mobility.

A zone-based load balancing approach in cellular networks was investigated in [26]. The authors divided the network into zones, with a congested macrocell and its neighbors in one tier, and picocells within the zone area in another. The proposed algorithm prioritizes users closer to the macrocell for association, reducing the potential for uneven load distribution across the macrocell. Simulation results showed that the proposed algorithm is effective in achieving balanced load distribution in heterogeneous networks. Furthermore, the authors in [27] proposed a load balancing technique called “Swap-based Load Balancing” (SLB) specifically designed for managing access points (APs) in 5G networks. The algorithm aims to outline the issue of disproportional user distribution association across small cells, which can create congested areas and overloaded APs. The results show that SLB, particularly with biasing, effectively reduces load imbalance between APs while simultaneously improving signal quality for users.

The study in [28] focused on how different HCP configurations can impact the network performance in 5G networks. They explored various system scenarios with considerations of different user mobility speeds. The evaluation of system performance considers various key performance indicators (KPIs) such as Reference Signal Received Power (RSRP), HO probability, HOPP, RLF, HO interruption time (HIT), and HOF. The simulations showed that there is a trade-off in the results gained from different systems, where using lower HCP settings gives a significant disadvantage by raising the HOPP rate more as compared with the higher HCP settings. At the same time, using lesser HCP settings achieved notable improvement compared to higher HCP settings regarding RLF for all mobile speed scenarios. The study concluded with the need for careful adjustment of HCP system settings considering other factors such as the mobile environment and use case. The authors in [29] proposed a load-balancing algorithm designed for 5G and future 6G networks, particularly those utilizing high-frequency millimeter waves. This algorithm builds upon the traditional method of choosing the best RSRP to establish a connection during the initial access phase. The authors simulated their algorithm in an urban microcell (UMi) scenario with a multi-beam next-generation base station. The simulation results show that this approach has promise for improving load balancing in high-frequency cellular networks.

A distributed load balancing algorithm for multi-radio access technology (multi-RAT) HetNets was proposed in [30]. It aims to address the unbalanced network load distribution caused by random small-cell deployment and user mobility. The algorithm considers the QoS requirements of users and the disparate capabilities of multiple RATs, such as different propagation delays, to balance the network load. The proposed algorithm achieves a balanced load distribution among cells in a finite number of iterations. It shows the network performance in terms of load distribution, throughput, and the number of users with satisfactory QoS, as shown through simulations. The authors in [31] proposed a multi-unmanned aerial vehicle (UAV)-aided mobile-edge computing (MEC) system to address the computational limitations of IoT devices. Their approach involves deploying multiple UAVs as MEC nodes to provide offloading services. To optimize UAV placement and load balancing, a differential evolution-based algorithm was employed, and the problem was modeled as a generalized assignment problem. Furthermore, a deep reinforcement learning (DRL)-based task scheduling algorithm was introduced to enhance task execution efficiency within each UAV. The work offers insights into UAV-based MEC system design, particularly in terms of load balancing and task optimization.

A simulation was used by [32] to investigate load-balancing self-optimization (LBSO) in 5G and 6G networks, focusing on the impact of different mobility speeds on optimization algorithms. Their findings underscore the significance of user location in enhancing handover control parameters, with the distance-based algorithm demonstrating performance in reducing probabilities of HOPP and RLF, and improving spectral efficiency. While their work provides valuable insights, this study extends these findings by concentrating on extremely dense UE deployment, enabling a more granular analysis of distance-based load-balancing strategies in this specific context.

Existing load-balancing techniques often have limitations in addressing highly dynamic user distributions, particularly in ultra-dense network environments like those encountered in urban centers or during large-scale events. These environments experience rapid changes in user density, leading to persistent congested cells in specific areas while others remain underutilized. Traditional methods designed for static or gradual user pattern changes might not effectively adapt to such dynamic conditions. To address this gap, this study proposes the Dynamic Distributed Load Balancing (DDLB) algorithm specifically designed to handle ultra-dynamic user distributions and optimize network performance.

## 3. System Model

This study is focused on optimizing network performance by utilizing MM mechanisms within an ultra-dense HetNet environment consisting solely of small cells, specifically microcells. These microcells work together to provide a seamless UE connection. UEs connect to cells based on their signal strength metrics RSRP or Reference Signal Received Quality (RSRQ) level. While this ensures basic connectivity, it can lead to inefficiencies in highly dynamic user distribution scenarios, particularly in ultra-dense network environments. In such environments, UEs prioritizing strong signals might overload certain cells, causing uneven user distribution and underutilized resources in less congested cells. This leads to uneven user distribution, with congested areas experiencing limited resources for UEs while others remain underutilized. Consequently, the overall network efficiency suffers. On the other hand, the traditional cell selection for HO processes relies solely on maximizing signal strength metrics (RSRP or RSRQ) too. This approach leads to several issues:

- Unnecessary HOs: UEs might unnecessarily switch to congested cells due to strong signal strength, even if their current connection is sufficient. This wastes resources and increases signaling overhead.
- Service Disruptions: Frequent and unnecessary HOs can trigger RLF or HOPP, further impacting user experience with dropped calls or delays.

Current load-balancing approaches often prioritize static network configurations and struggle to adapt to real-time changes in user density. This can lead to persistent congestion in specific areas while other areas remain underutilized. Furthermore, a cell with an overloaded state is a critical challenge within this context. When a cell experiences a surge in user traffic or insufficient resources to handle existing traffic, it becomes overloaded. The duration of this overload significantly impacts network performance. Prolonged overload can lead to a domino effect, as UEs struggling to connect to congested cells might put additional strain on neighboring cells, further escalating the issue. These limitations in cell selection, handover procedures, and load-balancing techniques can lead to significant inefficiencies and a degraded user experience within the network. Users might experience the following:

- Reduced Network Throughput: Congestion can limit the overall data transfer rate experienced by users.
- Increased Latency: Delays in data transmission due to congestion and unnecessary HOs.
- Higher Call Drop Rates: Increased likelihood of dropped calls due to RLF or HOPP triggered by inefficient HOs.

The next section will introduce the DDLB algorithm, specifically designed to address the limitations identified in the current system model. The DDLB algorithm aims to improve overall network performance by dynamically adjusting target cell selection criteria based on real-time network conditions, particularly congestion levels.

### Proposed Dynamic Distributed Load-Balancing Algorithm

**Figure 2:**
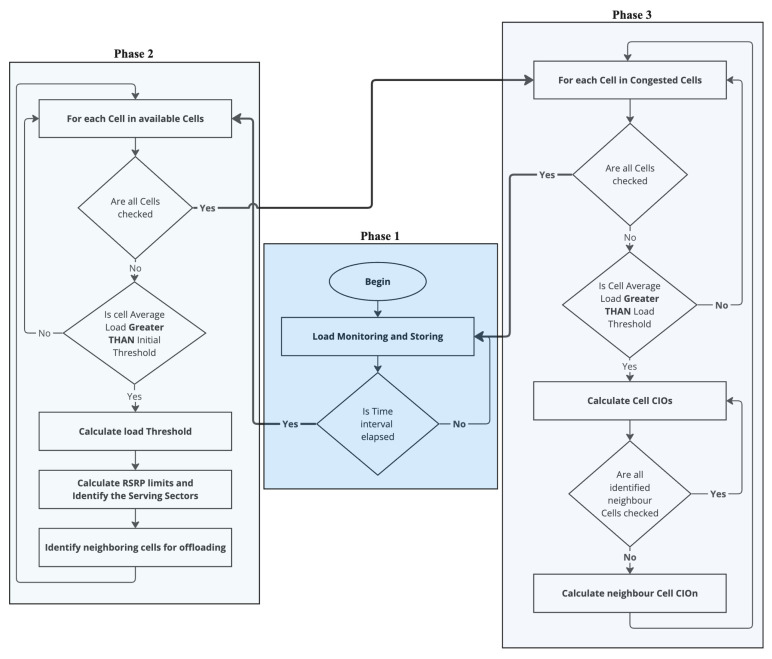
The algorithm block diagram.

In order to address limitations associated with cell congestion and inefficient load balancing in ultra-dense networks, this study develops the DDLB algorithm. By leveraging the MM mechanisms to enhance network performance, the DDLB algorithm can efficiently distribute network load across contiguous cells within a HetNet deployment. The DDLB outcomes contribute to improved processes of HO triggering, target cell selection, and decision-making for each UE according to their location and the load condition on nearby cells. The algorithm performs a flexible adjustment on the criteria of finding suitable target cells. This adjustment changes dynamically over time to adapt to the data traffic conditions of the users in specific locations as shown in Figure 2. This prioritizes the neighboring cells with higher resource availability to be selected to transfer the UEs connection, considering the locations of the UEs to the neighboring cells and the signal power to grant the best service quality.

**Figure 3:**
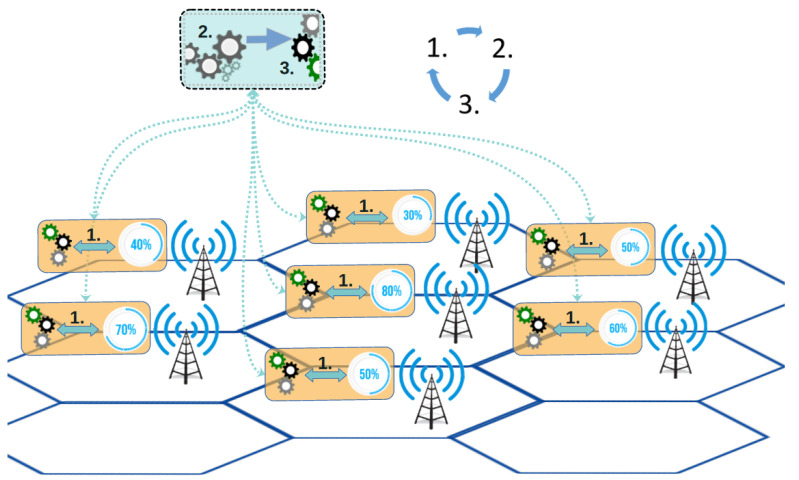
Architecture of Dynamic Distributed Load-Balancing algorithm.

**Figure 4:**
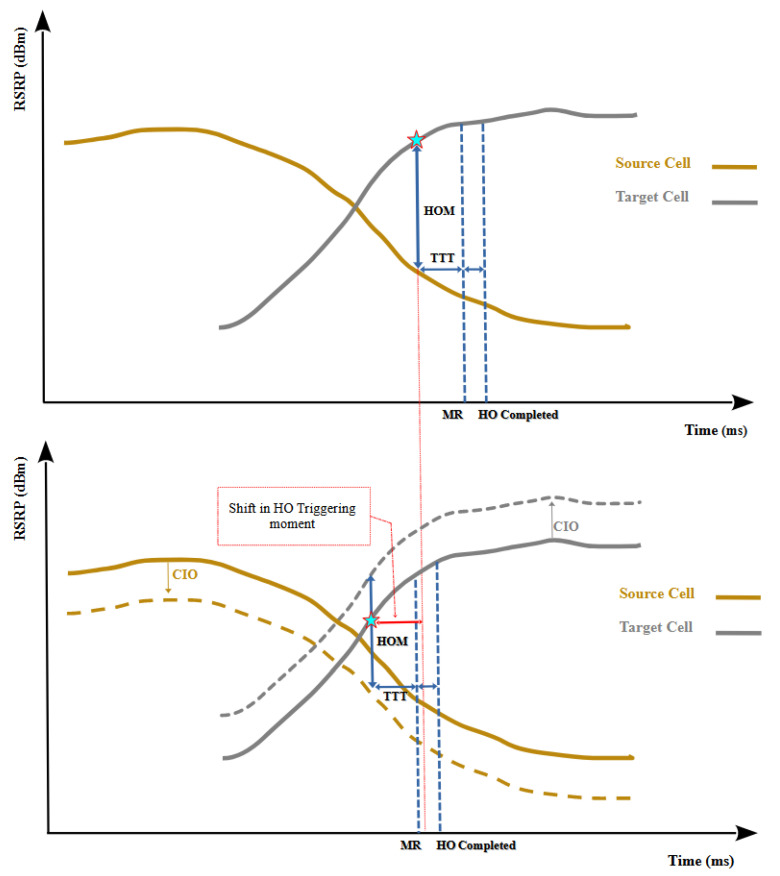
CIO Influence on HO triggering moments: CIO is a the parameter that affects the received signal power levels of individual cells when the UE performs signal quality checks. The CIO values modify the received signal power thresholds, influencing the moments when handovers occur.

To achieve these functionalities, the DDLB algorithm operates in three logical phases, as shown in Figure 3, that work together to continuously monitor network conditions, dynamically adjust load-balancing parameters, and influence handover decisions:

1. Continuous Load Monitoring: This phase functions continuously by collecting load fluctuation metrics for each cell. Every second, the DDLB algorithm stores data on the real-time load conditions experienced by each cell. This continuous monitoring provides the DDLB algorithm with a constantly updated status of current network traffic distribution. This real-time information is crucial for the DDLB algorithm to make informed decisions in the subsequent phases. In this context, the metric used to represent “load” can be the number of resources utilized.
2. Periodic Load Balancing (with Dynamic Interval): This phase operates periodically with specific intervals ($T_{interval}$). These intervals are adjustable dynamically based on network conditions typically measured in seconds. A shorter interval is chosen during periods of high congestion or rapid user density changes to ensure the DDLB algorithm remains responsive to network dynamics. Conversely, a longer interval can be used during stable network conditions to reduce signaling overhead. Network conditions for selecting the proper $T_{interval}$ can include factors like congestion times, user density in specific areas, the rate at which cells enter an overloaded state, and other relevant metrics. Within this phase, the DDLB algorithm performs three key tasks:
  - Congested Cells Identification: The algorithm first identifies cells that are currently experiencing high load, exceeding a predefined threshold ($Thresh_{initial}$). These congested cells are considered potential candidates for load-balancing actions.
  - Flexible Load Threshold Determination: For each loaded cell, the algorithm determines a flexible load threshold denoted by $load_{Thresh}$. This threshold is dynamically adjusted based on the average load conditions of its neighboring cells. This dynamic approach ensures the DDLB algorithm adapts to variant network traffic patterns.
  - Determination Neighboring Cell for CIO Updates: The DDLB algorithm prioritizes updating CIO values for surrounding cells that can potentially receive offloaded UEs from the loaded cell. This determination leverages the concept of cell sectors where each sector covers a directional area.
3. Cell Individual Offset (CIO) Determination and Update: Following phase 2, this phase performs the necessary calculations to determine the proper values of CIO for the congested cells and their neighboring cells. The employments of dynamic CIO values can accelerate or delay the HO triggering moment as shown in Figure 4. As a result, the coverage area of each cell is affected by either an extension or a reduction dynamically, depending on the new CIO value.

Then, the DDLB algorithm updates the determined CIO values in each loaded cell and in its neighboring cells that are identified as offloading targets. Following the continuous load monitoring in Phase 1, when the predefined interval $T_{interval}$ is reached, the DDLB algorithm identifies congested cells by comparing their average load ($load_{Av}$) to a predefined threshold ($Thresh_{initial}$). The $load_{Av}$ is calculated based on the load records collected during the $T_{interval}$ using the following equation:

(1) $$load_{Av}=\frac{\sum_{i=1}^{m}load\left(i\right)}{m},$$

where *m* is the number of stored load records of a cell during the period of $T_{interval}$. For each identified loaded cell, the DDLB algorithm then determines a flexible load threshold ($load_{Thresh}$). It considers the average load condition calculated based on the load condition of the loaded cell with its neighboring cells. For each identified loaded cell, the determination for load threshold ($load_{Thresh}$) is initiated as follows:

(2) $$load_{Thresh}=\frac{\sum_{i=1}^{n}load_{Av}\left(i\right)}{n},$$

where *n* is the number of cells, including the loaded cell and surrounding cells. This $load_{Thresh}$ acts as a trigger to determine the number of utilized PRB required to be released $load_{Rel}$ for offloading UEs from the congested cell toward the neighboring cells according to the following:

(3) $$load_{Rel}=\max\left(load\left(i\right)-load_{Thresh}\left(i\right),0\right).$$

Following the determination of $load_{Rel}$, the DDLB algorithm identifies the UEs suitable for offloading and their serving sectors and calculates the CIO values for both the congested cell and its neighboring cells in the direction of identified sectors. This process involves the following steps:

- UE Selection for Offloading: The congested cell requests the connected UEs to report the received signal strength (RSRP) for both the serving cell (congested cell) and neighboring cells. By analyzing the reported RSRP values, the DDLB algorithm identifies the UEs at the edge of the cell. These UEs are characterized by a low RSRP value from the serving cell (serving cell), and in the meantime, the difference in RSRP value between the serving cell and neighboring cells is low, too. The selection process prioritizes UEs with the worst serving RSRP value and a lower RSRP difference. This prioritization ensures UEs on the edge are offloaded first. The selection process stops when the cumulative load values of the selected UEs reach the $load_{Rel}$ and stores $RSRP_{limit}$ obtained from the RSRP value of the last selected UE. The selection process can also incorporate other factors beyond proximity based on RSRPs. These factors might include offloading of the service type based on the 5G QoS Identifier (5QI) or special conditions criteria in the neighboring cells reported by the UE (e.g., restricted access or blacklisted cells).
- Directional Awareness of Neighboring Cells: By considering the direction of cell sectors containing UEs selected for offloading, the DDLB algorithm identifies neighboring cells in those directions. This avoids unnecessary signaling overhead and focuses adjustments on the most relevant parts of the network. This targeted approach ensures CIO updates influence handover decisions for UEs at the cell edge, facilitating efficient offloading and promoting network performance.
- CIO Calculation: Once the UEs for offloading are selected and neighboring cells for CIO calculations are identified, the DDLB algorithm calculates the CIO values for both the congested cell ($CIO_{s}$) and its identified neighboring cells ($CIO_{n}\left(i\right)$). The $CIO_{s}$ value for the congested cell is determined using the following equation:
  (4) $$CIO_{s}=event_{Thr}-\left(RSRP_{limit}+Hys\right),$$
  where $event_{Thr}$ is the predefined RSRP threshold used for triggering the HO process. Hys is the hysteresis value. The $CIO_{n}\left(i\right)$ value for each neighboring cell(*i*) identified during the selection process is calculated using the following equation:
  (5) $$CIO_{n}\left(i\right)=\left\{\begin{array}{cc} |CIO_{s}+\beta \left(i\right)| & \mathrm{if}\;\beta \;>\;0,\\ |CIO_{s}-\beta \left(i\right)| & \mathrm{otherwise}, \end{array}\right.$$
  where β(i) is a factor that adjusts the offset for neighboring cell(*i*) which can be calculated using an equation like the following:
  (6) $$\beta \left(i\right)=RSRP_{limit}*\sigma -event_{Thr}\left(i\right)$$
  where the Scaling Factor (σ) as presented in Table 1 is represented by a value that varies based on the different network deployments. It ensures the CIO values do not cause the effect of HOPP. The $event_{Thr}\left(i\right)$ is the RSRP threshold used for triggering the HO process at the neighboring cell(*i*). The CIO values for neighboring cells are limited based on their load condition. This limitation ensures that overloaded neighboring cells do not receive a large number of offloaded UEs.

By following these steps, which are presented in Algorithm  1, the DDLB algorithm selects UEs for offloading from the congested cell and calculates the appropriate CIO values for both the congested cell and its identified neighboring cells. This facilitates the handover process and helps distribute the network load more efficiently.
**Algorithm 1:** Dynamic Distributed Load-Balancing (DDLB) Algorithm
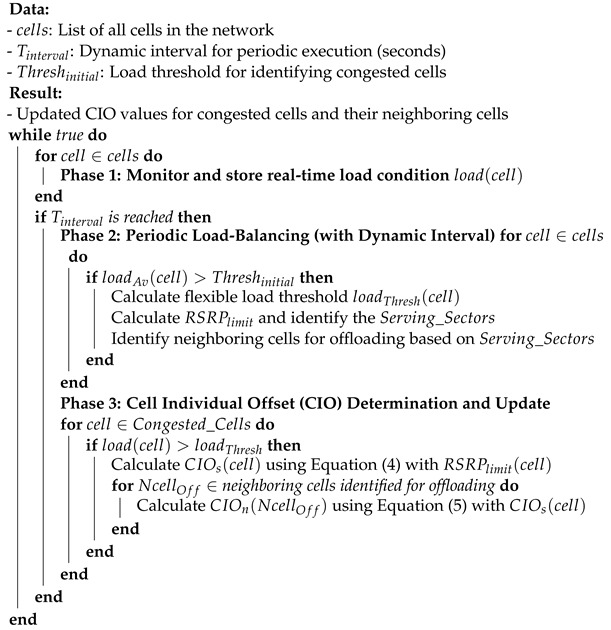


## 4. Simulation Scenario with Ultra-Dense Urban Environment

**Figure 5:**
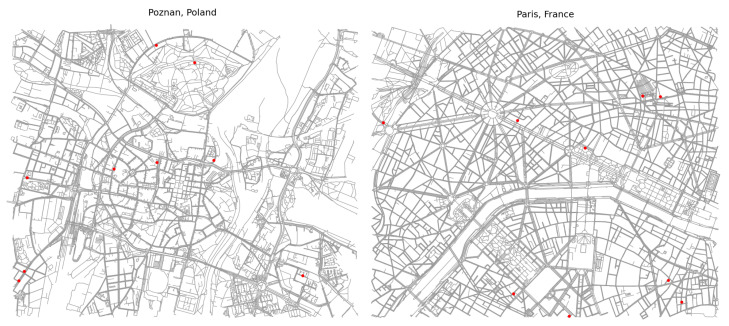
Layout of HetNet simulator environments.

In this research, a Python-based HetNet simulator was modeled based on 5G Evolution 3GPP Rel.17 specifications. The simulator allows for the creation of diverse network environments, including rural, urban, and suburban scenarios. It supports both macrocells and microcells that are operating in sub-6 GHz and mmWave frequency bands. The UE mobility is incorporated, allowing for UEs to move randomly within the simulated environment layouts, connect to neighboring cells, and perform the handover process based on A1, A2, and A3 events. The simulator automatically generates any specific area layouts based on its coordinates. Then, it distributes the UEs uniformly within this area as shown in Figure 5. Moreover, it accurately captures the network’s dynamic processes, simulating processes at the milliseconds level. This enables the evaluation of network performance metrics, particularly under different conditions such as HetNet implementation and a high user density. Thus, the evaluation highlights the effectiveness of the MM metrics such as HO, HOF, RLF, and HOPP rates and the impacts of network load distribution among the cells in a dense urban environment.

**Figure 6:**
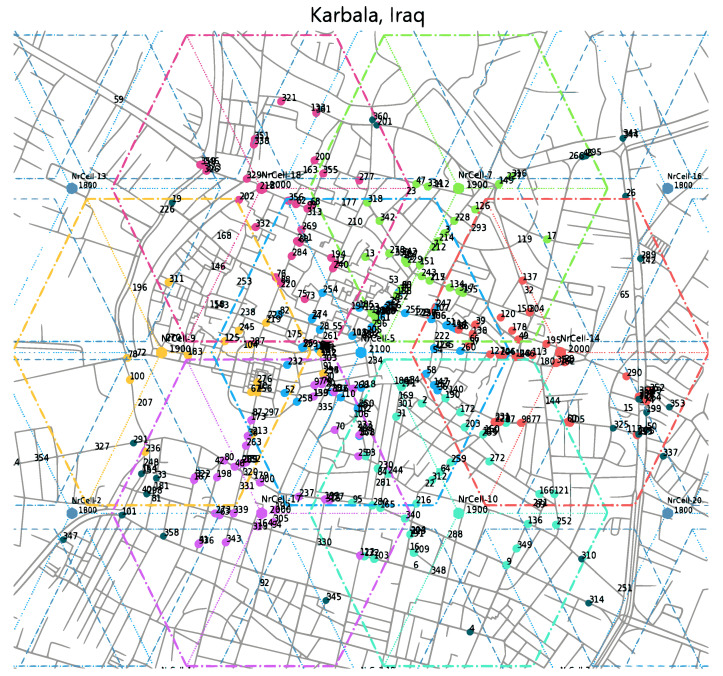
Network deployment scenario with cells and connected users.

Each hexagonal-shaped cell is divided into three 120° sectors for further network efficiency. This configuration ensures user location awareness, improves the connectivity of users, and enhances system performance. These cells operate at different carrier frequencies and channel bandwidths to minimize the effects of interference. The radius of each cell can vary based on cell type; macro-cells typically range from 500 m to 1200 m, while micro-cells cover 200 m to 500 m. The mobile users are spawned at random coordinates, destinations, and speeds. The speeds vary based on the simulated environment (e.g., city center: 3 to 20 km/h). To represent a real network environment, each user can stop for a random duration and then resume moving until reaching the simulated environment’s edge. Otherwise, the user is removed and a new one with a new configuration is spawned. Additionally, at regular intervals, several users are created with random edge coordinates, potentially entering the central area. The simulation scenario focuses on an ultra-dense urban environment, specifically the city center of Karbala, Iraq. Karbala shows significant seasonal fluctuations in user density patterns over the year. Additionally, the city hosts annual large religious gatherings that last for a few weeks, during which visitor numbers reach their peak with millions of visitors. This causes significant congestion that impacts all infrastructure, particularly wireless networks. To reflect this real-world scenario with resource limitations, the simulation deploys a limited number of UEs (around 400) concentrated in the simulated area. Some UEs with low-speed rates simulate co-located pedestrians using a pedestrian mobility model. The user data traffic model incorporates various data rates between 15 Mbps and 35 Mbps, reflecting typical usage patterns and potential network congestion. Therefore, seven 5G cells (differently colored as shown in Figure 6) are located centrally to evaluate the performance of the DDLB algorithm. Most users are required to pass through the center of the simulated environment to allow for the consideration of various HO scenarios as well as additional possibilities with different load situations among the chosen cells, especially the center cell. Being aware of all limitations related to simulation-based experiments, we suppose that the considered scenario (Karbala city) could be treated as a good representative of other similar locations where an extremely high density of users can be noticed.

Table 1 summarizes the main parameters used in the simulation scenario. In each simulation cycle, users move toward their destinations. If a data session exists, the user checks various events related to the session state such as disconnection time, channel quality, RSRP, and MM events. Otherwise, if it is time to establish a data session, the UE searches for the best nearby cell (i.e., with the highest RSRP value) and sends an attach request to establish a data session. Rejected attach requests for any reason (e.g., all resources are reserved) lead to updating the attach failure counter of the cell that rejects the request, and attempts are made with other suitable cells. UE with an established data session is visualized by circles colored according to the serving cell color. Each cell updates its data rate, resource utilization array, and load status. For users with established data sessions, several factors are considered to estimate signal strength such as line-of-sight (LoS), non-line-of-sight (NLoS), and log-normal shadowing. These factors influence the calculation of the RSRP and SINR values for each carrier signal.

Moreover, the configured MM events are checked as follows: if a UE meets A1/or A2 conditions, it sends an A1/or A2 MR to the serving cell. Similarly, if the A3 event is configured, the UE measures and stores RSRP values for neighboring cells within a specific time frame (TTT). Upon reaching TTT, the average RSRP from stored RSRP values of each neighboring cell is checked with A3 conditions. If any cell meets the condition, it is included in the A3 MR with its average RSRP value. Upon reception of A1 or A2 MRs, the serving cell evaluates the handover necessity. Where A1 MR indicates that the UE has a good signal stat ($RSRP_{s}+Hys>event_{Thr}$), the cell proactively responds with a request to remove the A3 event, if present, to avoid unnecessary measurements. Conversely, for an A2 MR that indicates that the UE has a poor signal stat ($RSRP_{s}+Hys<event_{Thr}$), the A3 event configuration is triggered as planned to identify a better target cell during the handover evaluation. Upon reception of A3 MRs, a cell selects the best target cell (excluding blacklisted ones) and initiates the handover process by sending an HO request. An HO command is sent to the UE if the request is acknowledged. Rejected requests lead to updating the HO-rejected counter and blacklisting the cell after exceeding a retry limit. The target cell keeps track of HO-rejected requests per UE. Once the UE receives the HO command, the UE disconnects from the serving cell and establishes the connection to the target cell. The UE monitors the new connection in case any RLF or HOPP occurs. If RLF occurs directly after HO for any reason (e.g., sudden signal degradation), the target cell updates the RLF counter and the UE reestablishes to the old cell. Otherwise, the cell updates the RLF counter and the UE attempts to re-attach to the same cell. If re-establishment is impossible, then the attach failure counter of that cell is updated, and UE tries to attach to the second nearby cell with a good RSRP value.

### 4.1. Performance Evaluation

A load of a cell indicates the utilization of the resources of the cell expressed as a percentage ratio of currently used Physical Resource Blocks PRBs to the total number of PRBs in that cell:

(7) $$Load(\%)=\frac{PRB_{Used}}{PRB_{Total}}\times 100$$

Handover Performance:

- Attach failure rate is the metric that measures the percentage ratio of attach attempts to a cell that fails to the total attach attempts. A high attach failure rate can indicate issues related to cell discovery, signal degradation during the attach attempt, congestion, etc.

(8) $$Attach\;Failure(\%)=\frac{Failed_{attachAttemps}}{Total_{attachAttemps}}\times 100$$

- Handover failure rate (HOF) is the metric that expresses specifically the percentage ratio of handover attempts that fail to the total number of HO requests received by a cell. This could be due to several reasons such as target cell rejection, issues with the HO signaling process, or sudden signal degradation during ongoing handover, etc.

(9) $$Handover\;Failure(\%)=\frac{Failed_{HandoverRequests}}{Total_{HandoverRequests}}\times 100$$

- Radio link failure rate (RLF) is the metric that expresses the percentage ratio of the unexpected termination of an established data session due to signal quality-related issues or resource availability-related issues.
  (10) $$Radio\;Link\;Failure(\%)=\frac{Failed_{RadioLink}}{Total_{SucssefulAttachAttempts}}\times 100$$
- Handover ping-pong rate (HOPP) is the metric that measures the percentage ratio of a UE that switches back and forth between two cells within a short period due to the close difference in signal strength between cells.
  (11) $$Handover\;PingPong(\%)=\frac{PingPong_{HandoverRequests}}{Total_{SuccessfulHandoverProcesses}}\times 100$$

Load-Balancing Performance

- Overload duration rate (OL) is the metric that provides insights into the percentage rate of the time that a cell enters an overloaded state. Ideally, this rate should be as low as possible to minimize user experience issues like call drops or reduced data rates and ensure efficient network resource utilization.
  (12) $$Overload\;Duration(\%)=\frac{Time_{Overloadstate}}{Time_{simulation}}\times 100$$

### 4.2. Results Discussion

In this research, the investigation was conducted on the system performance under various user density patterns by employing a simulation framework. In each simulation, three separate environments were simulated with identical network configurations and similar user distribution. They vary in how the DDLB algorithm was applied as follows:

1. Environment Without Algorithm: represents the network’s performance without implementation of the DDLB algorithm. This is employed as a benchmark for comparison.
2. Algorithm 1-min Interval: represents the DDLB algorithm with an execution interval of 1 min. The algorithm performs its functionality every minute within the simulation.
3. Algorithm 3-min Interval: represents the DDLB algorithm that performs its functionality with a less frequent execution interval of 3 min.

**Figure 7:**
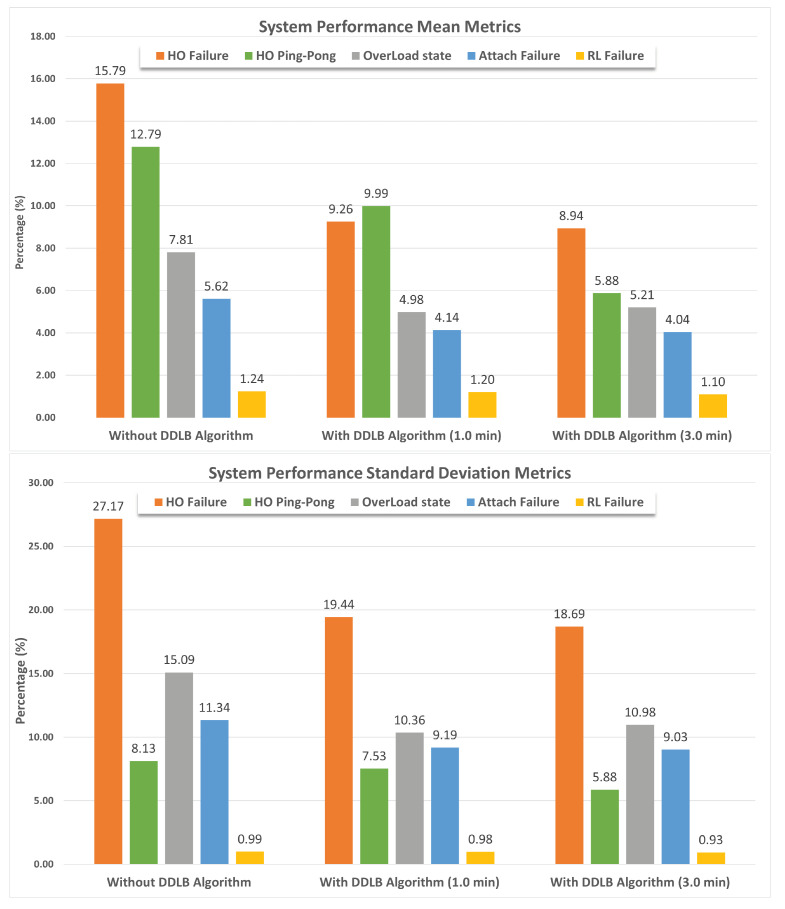
Metrics of mean and standard deviation of system performance.

By utilizing the same network configurations and user distribution across all three environments within a simulation, the impact of the DDLB algorithm and its execution frequency on network performance are effectively isolated. These controlled settings allow for a robust evaluation. The results presented in Figure 7 represent the percentage of mean and standard deviation values of system performance metrics obtained from the tested cells initially determined. The system performance metrics comprise various aspects such as HOF, HOPP, overloaded state, attach failure, and RLF calculated across 56 simulations. Each metric is independently measured for each cell at each simulation step. Then, the average and standard deviation are obtained for all cells. Each scenario was simulated for over 550k steps, where each step represents a millisecond.

**Figure 8:**
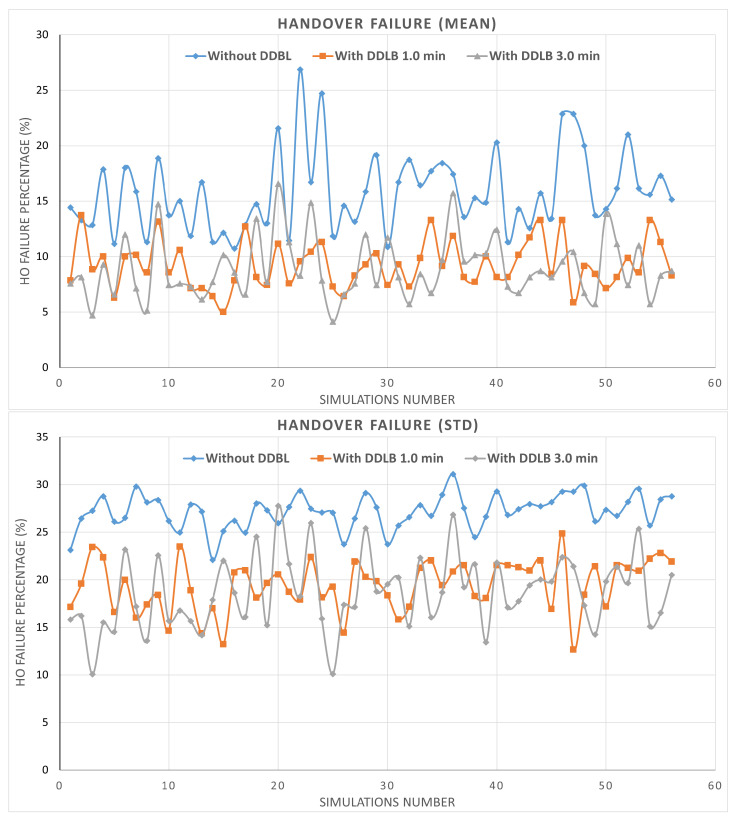
Handover failure ratio during all simulations.

The mean metric of HOF exhibits the highest rate at an average of 27.17% for the environment without the DDLB algorithm across all simulations as shown in Figure 8. This signifies a relatively high number of failed handover attempts. Utilizing the DDLB algorithm shows a significant reduction in the HOF rate. However, using the DDLB algorithm with a 1-min execution interval achieved an HOF rate of 19.44%, representing a 28.47% improvement compared to the environment without the algorithm. Utilizing the DDLB algorithm with a 3-min execution interval achieved an HOF rate of 18.69%, which represents a 31.21% improvement compared to the environment without the algorithm and 3.86% compared to the 1-min interval. This indicates that the algorithm effectively alleviated handover failures, demonstrating that the DDLB algorithm can improve handover success rates substantially.

**Figure 9:**
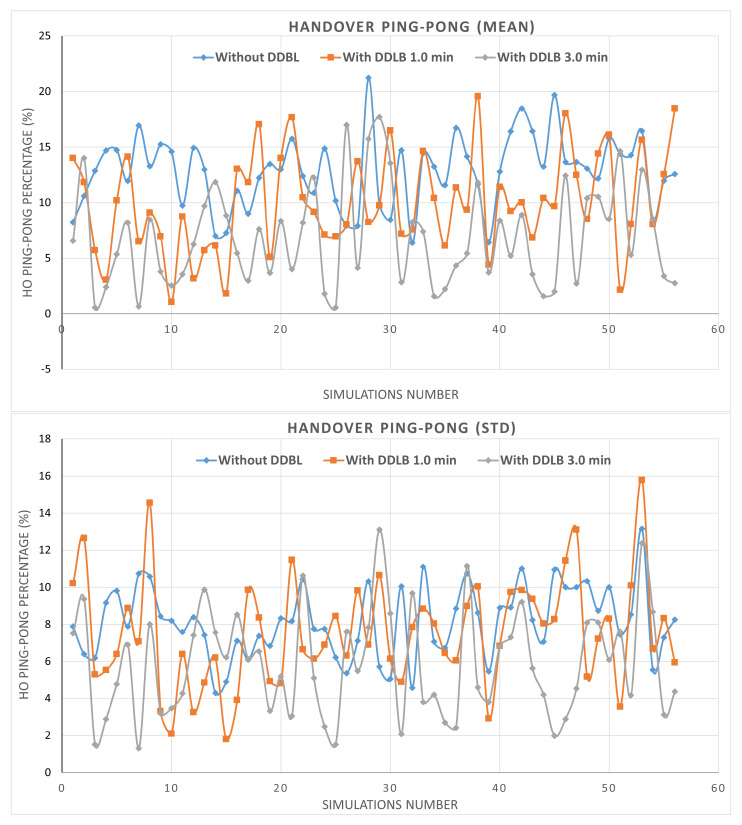
Handover ping−pong ratio during all simulations.

Next, the mean metric of HOPP also shows a high rate at an average of 12.79% for the environment without the DDLB algorithm across all simulations as shown in Figure 9. Utilizing the DDLB algorithm shows a reduction in the HOPP rate where the DDLB algorithm with a 1-min execution interval achieved an HOPP rate of 9.99%, which represents a 22.02% improvement compared to the environment without the algorithm. While utilizing the DDLB algorithm with a 3-min execution interval achieved an HOPP rate of 5.88%, which represents a 73.3% improvement compared to the environment without the algorithm and 41.32% compared to the 1-min interval.

**Figure 10:**
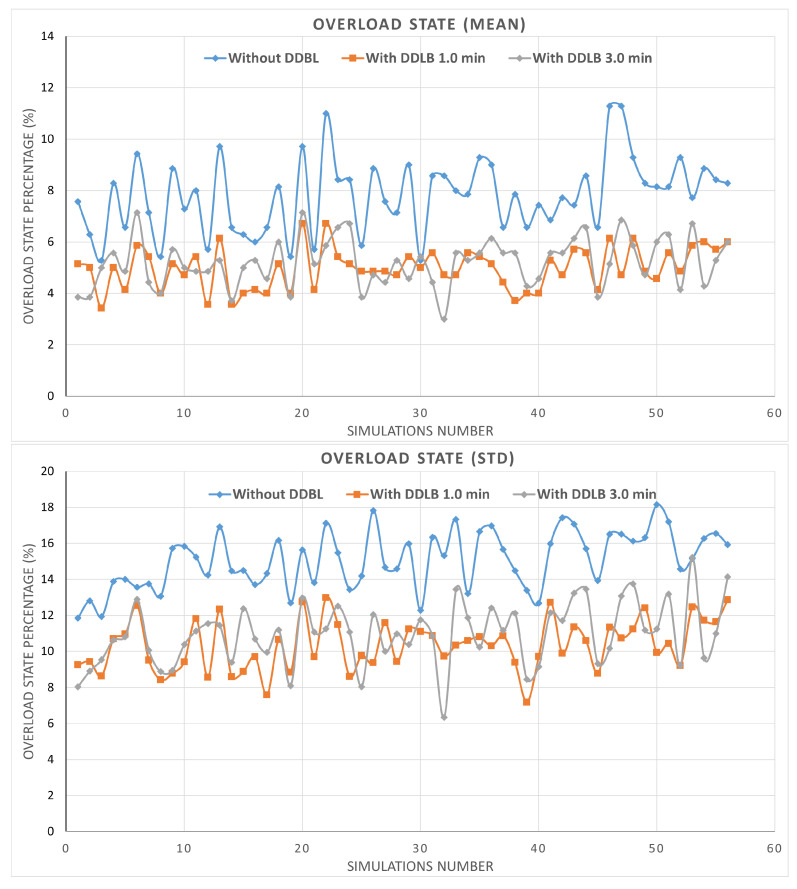
System overloaded state ratio during all simulations.

The OL state is a critical factor that can affect the performance of the network. The mean metric of the OL state also shows an effective reduction compared with the mean value achieved by the environment when the DDLB algorithm was not utilized. The improvement with the DDLB algorithm with an execution interval of 1 and 3 min achieved 36.24% and 33.29%, respectively, as shown in Figure 10.

**Figure 11:**
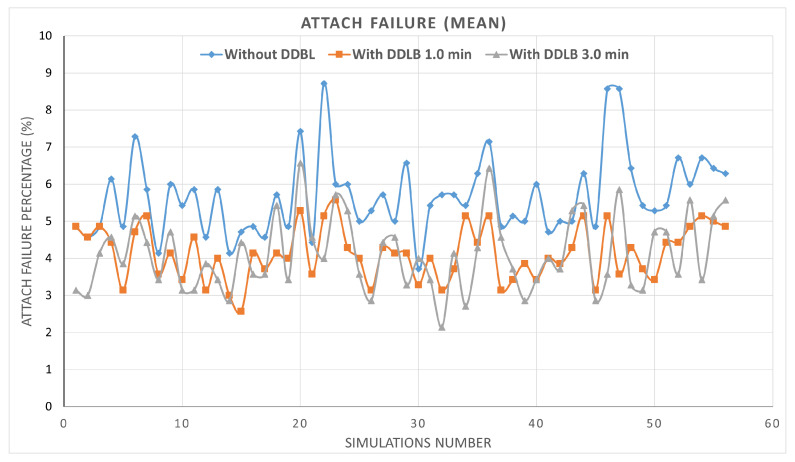
Attach failure ratio during all simulations.

The DDLB algorithm also demonstrated a positive impact on the mean metrics of attach failure and radio link failure (RLF). As depicted in Figure 11, the environment without the DDLB algorithm shows an attach failure rate of 5.62%. The DDLB algorithm reduced this rate significantly, with the 1-min execution interval achieving an improvement of 8.54% and the 3-min interval achieving an improvement of 27.94%.

**Figure 12:**
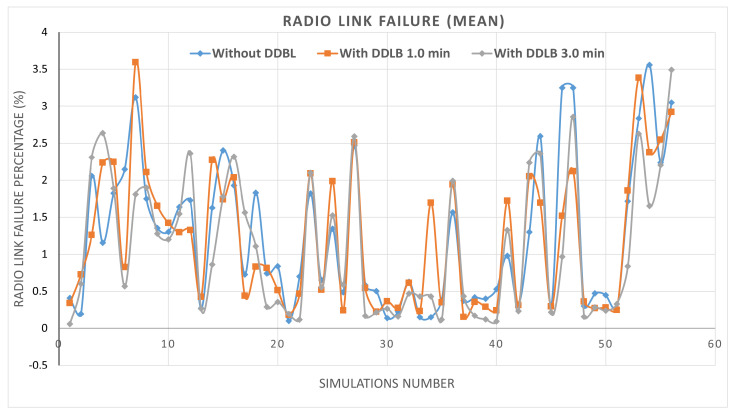
Radio−link failure ratio during all simulations.

Furthermore, the environment without the DDLB algorithm achieved a relatively low mean value of RLF of 1.24%. Interestingly, the DDLB algorithm with both execution intervals of 1 and 3 min maintained these low mean values of RLF with slight variations of 1.20% and 1.10%, respectively, as shown in Figure 12.

## 5. Conclusions

This study investigated the effectiveness of a DDLB algorithm in improving wireless network performance under unique user distribution patterns by employing a simulation framework, utilizing 56 simulations with varying user distributions to perform a wide range of network traffic patterns. The DDLB algorithm was implemented with different execution intervals (1 min and 3 min) within each simulation environment, allowing for a controlled evaluation of its impact.

The results showed significant improvements across several performance metrics. The DDLB algorithm mitigated HOF and HOPP occurrences effectively. Furthermore, the possibility of more benefits was observed even when a high execution interval (3 min) was utilized compared to a lower interval (1 min). This suggests that the algorithm can maintain effectiveness even with a lower computational overhead. Additionally, the algorithm exhibited a reduction demonstrable in the network overloaded state, with a marginally smaller impact observed with the utilization of the 1-min interval compared with the 3-min interval. Finally, the DDLB algorithm reduced the attach failure rates significantly through improved resource management and call admission control while maintaining stable radio link failure (RLF) rates.

In conclusion, this study showed that the DDLB algorithm offers a promising approach for enhancing a wireless network performance across different load conditions and user distributions. The algorithm’s effectiveness in mitigating both handover failures and network overload and optimizing attach success rates, even with a higher execution interval, suggests its potential for practical implementation with a balance between computational efficiency and performance benefits. Future studies could explore further optimization of the algorithm’s parameters and investigate its impact on other network performance metrics.

## 6. Future Work

While this research primarily focused on developing the DDLB algorithm for efficient load balancing within a microcell environment, there are several promising pathways for future investigation. Therefore, the integration of microcell–macrocell coordination is essential to further enhance the DDLB’s performance and applicability. By considering the interplay between these cell types, the DDLB algorithm can be extended to more heterogeneous networks. Additionally, investigating the potential benefits and challenges of continuous load balancing compared to the periodic approach adopted in this work is a promising avenue for future research. This could enable more dynamic and adaptive load management, potentially leading to improved system performance and user experience.

## Figures and Tables

**Table 1:** Simulation parameters.

Parameters	Values
Cell Type	5G cell
Number of Cells	24
Number of tested cell	7
Number of Users	385 Concentrated UEs, each UE simulates a group of UEs reflecting ultra dense occasions
Simulation Steps	530k step
Number of simulations	56
Transmit Power [dBm]	15
Antenna Gain [dBi]	12
Carrier Frequency [GHz]	1.8, 1.9, 2.0, 2.1
System Bandwidth [MHz]	25
Cell Radius [m]	450
BS Antenna Height	10
Resource Distribution	Distributed as user needs across all active UEs
UE Height [m]	1.5
UE Speed [km/h]	[3 to 20]
Mobility Model	non-uniform random mobility
Thermal Noise [dBm]	1.380649 × $10^{-23J/K}$ × Δf
Shadow Fading Model	A Gaussian-distributed random variable with zero mean and $\sigma_{dB}$ standard deviation in dB
Path ${\mathrm{Loss}}_{UMi-LOS}$ [dB]	32.4 + 40 $log_{10}$(*d*_3*D*_) + 20 $log_{10}$($f_{c}$) − 9.5 $log_{10}($(*d*′_*BP*_)^2^ + ($h_{UT}$ − $h_{UT}$)^2^)
Path ${\mathrm{Loss}}_{UMi-NLOS}$ [dB]	22.4 + 35.3 $log_{10}$($d_{3D}$) + 21.3 $log_{10}$($f_{c}$) − 0.3 ($h_{UT}$ − 1.5)
Shadowing STD [dB]	LoS = 4, NLoS = 7.82
A1/A2 Threshold [dBm]	−40.5
A1/A2 Hysteresis [dBm]	1.75
CIO Range [dBm]	[−20 to 20]
TTT values [ms]	360, 450
$Thresh_{initial}$	53%
$T_{interval}$ [s]	60, 180
Scaling Factor (σ)	1.07

## Data Availability

The data presented in this study are available on request from the corresponding author if not confidential.
